# Supplementary material for: MetaRibo-Seq measures translation in microbiomes
Source: Nat Commun. 2020 Jun 29;11:3268. doi: 10.1038/s41467-020-17081-z (PMC7324362; doi:10.1038/s41467-020-17081-z)
Supplement: Supplementary file 10 — Supplementary Data 7 [file 41467_2020_17081_MOESM10_ESM.zip › File2/Confidence_High_Taxonomy/89435_out.krona.html]

Javascript must be enabled to view this page.

members
magnitude
magnitudeUnassigned
count
unassigned
taxon
rank

89435\_out

3


SRS042628\_contig\_number\_16989
1

2
superkingdom
2

2
phylum
1224

28216
2
class

80840
2
order

2
family
119060

genus
2
1822464

1701573

SRS018351\_contig\_number\_contig-100\_10644.10644SRS143876\_contig\_number\_contig-100\_13033.48807
2
species
